# Permanent Rooms for the Buffalo Medical Association

**Published:** 1863-05

**Authors:** 


Permanent Rooms for the Buffalo Medical Association.—We are informed by Prof. White, the delegate appointed to procure assignment of permanent rooms for the Buffalo Medical Association, in the new building proposed to be erected for the Young Men's Association, Grosvenor Library, Law Library, and various Historical and Scientific associations, that the application was very favorably received, and no doubt could be entertained that the wishes of the Association would be gratified. Especially was this proposition regarded with favor, when the Committees were reminded that this society was the oldest scientific association in the City, and for years the only living, active organization having for its object the promotion of any science or profession; that its transactions had been published monthly for many years, and regarded by the profession everywhere as exceedingly interesting and valuable.

